# Impulsive and Reflective Processes Related to Alcohol Use in Young Adolescents

**DOI:** 10.3389/fpsyt.2014.00056

**Published:** 2014-05-22

**Authors:** Sara Pieters, William J. Burk, Haske Van der Vorst, Rutger C. Engels, Reinout W. Wiers

**Affiliations:** ^1^Training and Performance Innovations, TNO, Soesterberg, Netherlands; ^2^Behavioural Science Institute, Radboud University, Nijmegen, Netherlands; ^3^Addiction, Development and Psychopathology (ADAPT) Lab, Department of Psychology, University of Amsterdam, Amsterdam, Netherlands

**Keywords:** implicit cognition, explicit cognition, adolescents, alcohol use, working memory capacity, dual process model

## Abstract

**Background:** Dual process models suggest that the development of addictive behaviors is the result of interplay between impulsive and reflective processes, modulated by boundary conditions such as individual or situational factors. Empirical support for this model has been repeatedly demonstrated in adult samples [for a meta-analysis, see Ref. (1)]. The purpose of this study was to test these processes as they relate to emerging alcohol use in adolescents. Specifically, the interactive effects of several measures of impulsive and reflective processes and working memory capacity (WMC) are examined as predictors of changes in alcohol use among adolescents. It was expected that measures of reflective processes would better predict changes in alcohol use than measures of impulsive processes. Moreover, it was anticipated that WMC would moderate the relation between alcohol-specific impulsive and reflective processes and changes in adolescent alcohol use.

**Methods:** The sample consisted of 427 adolescents (47.7% male) between 12 and 16 years of age (*M* = 13.96, SD = 0.78) who reported drinking alcohol at least once. Four measures of impulsive processes were included. Attentional bias for alcohol was assessed with a Visual Probe Test; approach bias toward alcohol was assessed with a Stimulus Response Compatibility (SRC) Test; and memory associations with alcohol were assessed with an Implicit Association Test (IAT) and a Word Association Test. Two measures of reflective measures were included: positive and negative expectancies. WMC was measured using a Self-Ordered Pointing Task.

**Results:** Results showed that positive expectancies predicted changes in alcohol use, but this effect was qualified by an interaction with IAT scores. Moreover, SRC scores predicted changes in alcohol use only when negative expectancies were low. Attentional bias and word association scores did not predict changes in alcohol use. The relations between alcohol-specific processes or reflective processes and alcohol use were not moderated by WMC.

**Conclusion:** Although there is empirical evidence for the validity of the model in predicting heavier alcohol use in adolescents, or alcohol abuse and dependence in adults, these observations do not generalize to a sample of normative, early adolescents. More specifically, results indicated that reflective processes are more important predictors of changes in alcohol use than impulsive process during adolescence.

## Introduction

Dual process models of addiction state that the development of addictive behaviors is the result of interplay between impulsive and reflective processes, modulated by boundary conditions such as individual or situational factors (2–4). Impulsive processes are automatic, relatively less conscious processes, while reflective processes are deliberate and conscious processes. Impulsive processes become stronger with repeated use due to associative learning and alcohol-induced neural sensitization, while the ability to reflect on behavior is negatively affected by drinking. Support for the applicability of elements of dual process models to various health behaviors, including addictive behaviors, in adults is accumulating [e.g., Ref. (3, 5, 6)]. However, to date much less is known about how these models relate to substance use and the development of addictive behaviors in adolescence. The main aim of the current study is to examine whether the development of young adolescent drinking patterns can be explained by (elements of) the dual process model.

It has been suggested that adolescents are inclined to prefer behaviors that involve a certain risk, such as experimenting with alcohol and other illicit drugs (7). Several models of adolescent brain development have emphasized that risky behaviors are the result of an imbalance in the development of two neural systems: on one hand, an affective system that develops swiftly in parallel with hormonal developments during puberty, and on the other hand a reflective system that matures relatively more slowly and does not reach maturity until young adulthood [e.g., Ref. (8–13)]. The former system is dependent on neural circuits encompassing the striatum/nucleus accumbens [and more limbic areas, cf. the triadic model of Ref. (10)], associated with affective and reward-driven processes, while the latter is associated with more lateral prefrontal regions (9). Imbalance in brain development during adolescence may explain why adolescents are less likely to inhibit the motivation to approach a potentially rewarding, yet risky, stimulus.

These theoretical models are in line with dual process models [e.g., Ref. (3)], suggesting that substances affect related developing neural systems, meaning that repeated alcohol use sensitizes neural systems associated with the attribution of salience to cues previously associated with alcohol use [e.g., Ref. (14)], while alcohol use impairs more lateral prefrontal regions associated with the reflective system. As a result, impulsive processes become stronger, leading to attentional and approach biases toward alcohol (cues), while the reflective system can no longer inhibit these automatic impulses.

To date, most research has focused on reflective processes, measured with questionnaires examining attitudes toward alcohol, expectancies from alcohol, and motives for drinking alcohol. Studies have shown that even before children engage in alcohol use, they already develop expectancies from alcohol (15–18). Before the age of 10, attitudes toward and expectancies about the effects of alcohol are predominantly negative (16–19). After the age of 10, there is an increase in positive expectancies. Whereas some suggest that negative expectancies are replaced by positive ones (17, 20), others suggest that positive and negative expectancies co-exist, with positive expectancies becoming stronger over time (15).

Studies have shown that positive expectancies predict alcohol use concurrently [e.g., Ref. (21)] and longitudinally [e.g., between 6 months and 9 years; (22–24)]. In addition, positive expectancies predicted alcohol use problems in adulthood (25). While many studies have examined positive expectancies and adolescent alcohol use, fewer studies have reported on negative expectancies. Some report that negative expectancies are negatively related to alcohol, such that the more negative expectancies adolescents have, the less they drink [e.g., Ref. (26–28)]. Other studies have not been able to demonstrate a relation between negative expectancies and levels of alcohol use [e.g., Ref. (29)].

Alcohol-specific impulsive processes are assessed using so-called indirect measures. For instance, implicit associations between concepts in memory are often assessed in paper-and-pencil tasks, where individuals are prompted to give their first association with ambiguous words (e.g., draft, which can be related to a manuscript or to beer) or in reaction time tasks such as varieties of the Implicit Association Task (IAT), where the relative strength between concepts in memory is thought to be reflected in reaction times (e.g., alcohol and positive vs. alcohol and negative). Attentional and approach biases can also be measured using reaction time paradigms in which the relative speed of responding to alcohol vs. neutral cues is measured.

The use of indirect measures, in addition to more direct measures, such as traditional surveys assessing expectancies from alcohol use, has several advantages. For instance, indirect measures tap processes that are less easily available to introspection than direct measures. Scholars have indicated that expectancies differ from associations in memory in several ways. For instance, brain networks that are associated with either process do not necessarily overlap to a large extent [e.g., Ref. (30–33)]. In addition, associations develop slowly, whereas expectancies can be formed or changed based on a single occasion (34). Expectancies are also related to a true or false statement, whereas associations are not (35). Furthermore, associations are bidirectional, while expectancies rely more on “if … then” relations (36). Although expectancies sometimes rely on a single association (e.g., drinking alcohol and fun), this is not always the case, as for instance in expectancies related to negative reinforcement (37). Whereas direct measures require conscious introspection, indirect measures tap into associations that influence behavior relatively automatically or unconsciously. Explicit and implicit cognitions have been found to predict a unique part of the variance in alcohol use [meta-analysis, Ref. (1)].

Empirical studies examining the relation between impulsive alcohol-related processes and alcohol use in adolescence are scarce. Regarding young adolescents, approach biases have been shown in drinkers compared to abstainers, especially in adolescents who were less able to inhibit behavior [*N* = 374; (38)]. In addition, approach bias predicted drinking when inhibition was low over a period of 6 months (39). Yet, the two studies by Peeters et al. (38, 39) investigated adolescents with externalizing problems enrolled in special education. These adolescents are thought to have a higher chance of an early onset of alcohol use or alcohol problems in the first place. Using a normative sample, Willem et al. (40) demonstrated that approach bias was related to alcohol use in older male adolescents (*N* = 94), with no significant moderation by inhibitory control. Attentional bias was associated with alcohol use, but only in those with low attentional control. Further, in a study among 100 12- and 15-year-olds, it was found that boys with stronger implicit positive associations with alcohol had higher levels of binge drinking (more than five glasses per occasion) 1 year later than those without these positive associations (41). In addition, Thush et al. (42) showed that in a sample of at-risk adolescents, implicit cognitions predicted prospective drinking, over and above the effect of explicit cognitions. Measures of implicit alcohol-related memory associations were shown to be the best predictor of prospective drinking (42). Van Der Vorst and colleagues recently showed that implicit alcohol-related memory associated predicted alcohol use 1 year later in 608 children who never drunk at baseline (43). In addition, Pieters et al. (44), using an IAT, found that children (*N* = 134) associated alcohol more strongly with negative, compared to positive faces. It is important to note that some studies fail to find the proposed relation between impulsive reactions (approach bias) and alcohol use (45). Studies in adolescents have also shown that when executive control is low, alcohol-related automatic processes drive behavior, while when executive control is high, the reflective system overrides [e.g., Ref. (42, 46)].

To date, research on the dual process model in adolescents has mostly focused on concurrent data, used short-term follow-up assessments, or had small and selective samples [e.g., Ref. (42, 46)]. In addition, most previous research has examined relations between impulsive *or* reflective processes and alcohol use while there is some evidence suggesting that impulsive *and* reflective processes contribute to the prediction of adolescent alcohol use [e.g., Ref. (38, 39, 42, 46)]. Thus, the main aim of the current longitudinal study is to examine conditional effects of alcohol-related impulsive processes (e.g., alcohol-related memory associations, attentional bias, and approach bias), reflective processes (i.e., positive and negative expectancies from alcohol use), and working memory capacity (WMC) (as a measure of executive functions) on changes in alcohol use in adolescents who drank alcohol on at least one occasion. WMC was used as an indicator of executive functions, since it has been used in previous research studying the dual process model in adolescents. While there is some debate as to the number and types of executive functions [e.g., Ref. (47–49)], the three functions that are mentioned most often are response inhibition, working memory, and task shifting [e.g., Ref. (48, 49)]. Since there is some overlap between these functions, this phenomenon is called the “unity and diversity of executive functions” (49). We expected that both alcohol-related impulsive processes and positive expectancies would be positively related to alcohol use. WMC and negative expectancies were expected to predict less alcohol use. In addition, it was hypothesized that, when WMC is low, alcohol-related impulsive processes predict alcohol use, while when WMC is high, expectancies would predict alcohol use over time.

## Materials and Methods

### Participants

The sample consisted of 427 adolescents (47.7% male) between 12 and 16 years of age (*M* = 13.96, SD = 0.78) who reported drinking alcohol at least once. Roughly equal numbers of participants attended college-preparatory (32.7%), intermediate or basic education (28.2%), and vocational education (28.9%) classrooms. A total of 10.1% did not provide information on educational track, which mainly consisted of first graders who had not yet made a choice.

### Procedure

Data were derived from the first two waves of a larger longitudinal project assessing risk factors related to adolescent alcohol use [see also Ref. (50–53)]. Initially, 1215 adolescents attending five schools and their parents were contacted by mail. A total of 725 adolescents (60%) returned consent forms signed by themselves and one of their parents or caregivers, and 556 (77%) participated in at least one of the annual assessments. Of the 556 participants, 129 reported never drinking alcohol, leaving a sample of 427 adolescents who reported drinking alcohol at least once. Participants performed several tasks on a computer, including a Visual Probe Test (VPT), a Stimulus Response Compatibility (SRC) task, an Implicit Association Test (IAT), and a Self-Ordered Pointing Task (SOPT). The following day, adolescents completed an extensive questionnaire assessing alcohol use and expectancies from alcohol use. Both sessions lasted about an hour and a trained research assistant was available in both sessions to ensure that adolescents completed the sessions individually. In addition, the trained research assistant guaranteed confidential handling of the information provided by the adolescents. This study was approved by the review board of the Medical Ethical Committee in The Netherlands.

### Measures

#### Alcohol use

Adolescents completed four items describing the intensity of alcohol use during weekdays, on the weekend, at home and outside home (54). Items were summed to create a measure of weekly alcohol consumption. Alcohol consumption was measured during both annual waves of the study.

#### Approach tendencies

An SRC task (55) was used to assess alcohol-related approach tendencies. In this task, an alcohol-related picture or a soft-drink-related picture was presented on the screen in a randomized order. A manikin was placed either above or below the picture. Participants were asked to simulate an approach or an avoid movement if the picture was alcohol-related by pressing the arrow pointing upward or downward dependent on the orientation of the manikin. The order of blocks was counterbalanced across subjects, meaning that some participants were first instructed to approach alcohol-related pictures and to avoid alcohol-related pictures in the subsequent block. Each of the two blocks consisted of 40 trials. An SRC score was calculated by subtracting the mean reaction times (correct trials) when approaching alcohol (and avoiding soda) from mean reaction times when avoiding alcohol (and approaching soda) and a positive score indicated faster responses when instructed to approach alcohol. On average, participants made 7.58 errors on this task (SD = 7.51). Scores from participants with more than 33.33% errors in this task were omitted from the analyses. This resulted in 10 omissions for the SRC. Furthermore, *Z*-scores were computed and scores more than three SD lower or higher than the mean were adjusted to the next lowest or highest value.

#### Attentional bias

A VPT was used to assess attentional bias toward alcohol-related cues. In this task an alcohol-related and a soft-drink-related picture appear simultaneously on a computer screen. After 1500 ms, a black screen was shown followed by an arrow pointing upward or downward in the location of either the alcohol or soft-drink-related picture. The arrow remained visible until the participant responded to it by pressing the corresponding arrow on the keyboard. Attentional bias is inferred if reaction times to arrows replacing alcohol pictures were faster compared to soft-drink pictures. For this study, we subtracted mean reaction times to arrows replacing alcohol-related pictures from mean reaction times to arrows replacing soft-drink-related pictures. A positive score indicated a relative attentional bias toward alcohol-related photos. On average, adolescents made 7.49 errors on the task (SD = 9.84). Scores from participants with more than 33.33% errors in this task were omitted from the dataset. This resulted in an 18 omissions for the VPT. Furthermore, *Z*-scores were computed and scores more than three SD lower or higher than the mean were adjusted to the next lowest or highest value.

#### Memory associations

An IAT and Word Association Test (WAT) were used to assess memory associations. The unipolar IAT was used to assess the strength of associations between “alcohol” and “positive arousal” in memory [see Ref. (42)]. In this task, participants were instructed to categorize pictures and words as quickly as possible in one of four categories displayed in the upper corners of a computer screen by pressing the “E” or “I” button on a keyboard. In the incompatible block, category labels of “alcohol” and “neutral” were placed in one upper corner of the screen, while category labels of “unrelated to alcohol” and “positive arousal” were placed in the other upper corner of the screen. In the compatible block, the pairings were changed so that “alcohol” and “positive arousal” shared one upper corner, and “unrelated to alcohol” and “neutral” shared another. The order of the compatible and incompatible blocks was counterbalanced across participants. A D2SD penalty score was calculated by subtracting mean reaction times in the compatible block from mean reaction times in the incompatible block, meaning that positive scores reflected relatively faster responses in the compatible block. Positive scores were assumed to reflect a relatively better association between “alcohol” and “positive arousal” compared to “alcohol” and “neutral” concepts in memory. On average, participants made 20.2% errors (SD = 11.35). Scores from participants with more than 33.33% errors in this task were omitted from the dataset. This resulted in an omission of 45 participants’ scores on the IAT. Furthermore, *Z*-scores were computed and scores more than three SD lower or higher than the mean were adjusted to the next lowest or highest value.

In the WAT, participants were asked to write down the first word that came into mind upon reading written verbal cues. Cues consisted of 24 Dutch homographs, of which 6 were related to alcohol. The amount of alcohol-related answers that were given was used as an outcome measure on this task of associative memory [e.g., Ref. (56)].

#### Working memory capacity

A computerized version of the SOPT was used to assess WMC. This task was based on the original SOPT (57). Participants were instructed to select a picture from a matrix of pictures in each trial. In the next trials, participants had to repeatedly select another picture, in another location. The arrangement of the pictures was changed in every trial. Four versions of the task were utilized: 9 concrete pictures (people performing various sports), 9 abstract pictures (grayscale images), 12 concrete pictures (pictures of farm animals), and 12 abstract pictures (grayscale images). In the case of nine pictures, there were nine trials. For each version, we calculated the percentage of correct trial, and we averaged these percentages over the four versions. Higher scores indicated better WMC. The psychometric properties of the SOPT are sufficient (58) and the task is easy to administer in adolescents. In the current sample, the internal consistency of the four versions was good (Cronbach’s alpha = 0.87). The SOPT has been used in previous studies testing hypotheses regarding the dual process model [e.g., Ref. (42, 46)]. Participants on average responded correctly in 77.20% of the trials (SD = 20.31). We adjusted the scores (% correct) of adolescents with scores lower than three SDs below the mean to the next available lowest value. They were considered to have not taken the task seriously, or to have an extremely insufficient WMC.

#### Alcohol expectancies

In a paper-and-pencil task, 12 statements were reported regarding the expected consequences (6 items for positive and 6 for negative expectancies) of alcohol use. All statements started by: “Drinking alcohol makes me” followed by a word indicating either a positive or negative consequence. For instance, “Drinking alcohol makes me happy” (i.e., cozy, comfortable, friendly, social, sympathetically, sad, moody, depressed, lonely, unhappy, down). Answers were given on a 10-point scale ranging from (1) not at all to (10) very much. The internal consistency of the scales was good (Cronbach’s alpha was 0.95 for positive and 0.94 for negative).

## Results

### Descriptives and correlations

Pearson correlations among all study variables are presented in Table 1. Gender was negatively correlated with negative expectancies, with males reporting more negative expectancies than females. Age was positively associated with wave 1 alcohol use and positive expectancies, with older adolescents reporting more drinking and more positive expectancies than younger adolescents. Education level was negatively associated with alcohol use at waves 1 and 2, with students in vocational classrooms reporting more alcohol use than those in university-preparatory classrooms. Alcohol use at wave 1 was negatively correlated with IAT scores, suggesting the more adolescents associate alcohol with arousal, the less they drink. Alcohol use at waves 1 and 2 was positively associated with alcohol-related memory associations and positive expectancies, with more alcohol-related memory associations and positive expectancies being associated with more concurrent and subsequent alcohol use. Positive expectancies were also positively associated with alcohol-related memory associations and negative expectancies. All other correlations were not statistically significant, including the associations between the four measures of impulsive processes.

**Table 1:** **Descriptives and correlations between study variables**.

	1	2	3	4	5	6	7	8	9	10	11	Mean	SD	Range
1. Gender														199 Boys, 228 girls
2. Age	−0.03											14.01	0.77	11–16 years
3. Education	0.07	−0.05												
4. Alcohol 1	−0.01	0.22***	−0.20***									1.52	0.82	1–6
5. Alcohol 2	−0.08	0.09	−0.19**	0.35***								1.79	0.98	1–6
6. Approach bias	−0.07	−0.03	−0.07	−0.05	0.11							47.35	851.36	-2988.5 to 3642
7. Attentional bias	−0.03	0.06	−0.08	−0.02	0.03	0.12						−3.13	36.25	-109 to 111.5
8. Memory associations (IAT)	0.04	0.01	0.07	−0.16*	−0.08	−0.02	−0.07					1.01	0.71	-2 to 4.13
9. Memory associations (WAT)	−0.06	0.16**	−0.00	0.20***	0.17**	−0.04	−0.07	−0.02				1.47	0.98	0–5
10. WMC	0.08	−0.04	0.12*	−0.04	−0.07	0.00	0.01	0.11	−0.01			77.04	20.19	9.72–100
11. Positive exp.	0.07	0.17**	−0.03	0.34***	0.26***	0.05	−0.02	0.02	0.29***	−0.03		4.67	2.72	1–10
12. Negative exp.	−0.12*	0.02	−0.19***	−0.02	0.02	0.05	0.04	−0.10	0.04	−0.01	0.22***	2.15	1.73	1–10

Gender is coded as 0 = male and 1 = female. WMC is working memory capacity. Positive exp. is positive expectancies. Negative exp. is negative expectancies; *p < 0.05, **p < 0.01, ***p < 0.001.

### Predicting alcohol use from alcohol-related impulsive processes and working memory capacity

Three multiple linear regression analyses were performed to identify predictors of changes in alcohol use from waves 1 to 2. In the first model, gender, age, educational level, the four impulsive processes (approach tendencies, attentional bias, and memory associations from the WAT and IAT), alcohol use at wave 1, WMC and four interactions between alcohol-related impulsive processes, and WMC were entered as predictors (see Table 2). Changes in alcohol use were used as a dependent variable in this analysis. Overall, the model explained 17% (*p* < 0.001) of the variance of alcohol use at wave 2. In this model, the only statistically significant predictor of alcohol use in wave 2 was alcohol use in wave 1. All main effects of WMC and the impulsive measures, as well as all four interaction terms were not statistically significant.

**Table 2:** **Results**.

	Model 1		Model 2		Model 3	
	β	*p*	β	*p*	β	*p*
Gender	− 0.053	0.277	− 0.063	0.211	− 0.075	0.145
Age	0.008	0.893	− 0.023	0.731	0.008	0.900
Level of ed.	− 0.103	0.129	− 0.084	0.258	− 0.050	0.609
Alcohol use 1	0.319	<0.001	0.330	<0.001	0.338	<0.001
SRC	0.115	0.232	0.036	0.694	0.159	0.088
VP	0.031	0.658	0.046	0.599	0.013	0.854
IAT	0.002	0.981	− 0.040	0.626	− 0.007	0.942
WAT	0.103	0.071	− 0.012	0.874	0.116	0.053
WMC	− 0.040	0.537	− 0.116	0.161	− 0.066	0.452
PE			0.148	0.002		
NE					0.059	0.453
SRC × WMC	− 0.020	0.833	0.051	0.686	− 0.146	0.201
VP × WMC	− 0.021	0.713	− 0.018	0.811	0.015	0.835
IAT × WMC	− 0.002	0.984	− 0.107	0.364	0.001	0.997
WAT × WMC	0.031	0.538	0.159	0.179	− 0.008	0.916
PE × WMC			0.063	0.452		
NE × WMC					0.022	0.763
SRC × PE			0.153	0.128		
VP × PE			0.019	0.837		
IAT × PE			0.199	0.042		
WAT × PE			0.053	0.385		
SRC × NE					− 0.238	0.035
VP × NE					0.006	0.946
IAT × NE					0.180	0.051
WAT × NE					− 0.066	0.466
SRC × WMC × PE			− 0.203	0.253		
VP × WMC × PE			− 0.033	0.732		
IAT × WMC × PE			0.052	0.628		
WAT × WMC × PE			− 0.079	0.423		
SRC × WMC × NE					0.087	0.557
VP × WMC × NE					0.118	0.295
IAT × WMC × NE					0.095	0.449
WAT × WMC × NE					0.045	0.558

*Gender is coded as 0 = male and 1 = female. SRC is Stimulus Response Compatibility Task. VP is Visual Probe Task. IAT is Implicit Association Test. WAT is Word Association Task. PE is positive expectances. NE is negative expectancies. WMC is working memory capacity*.

### Predicting alcohol use from alcohol-related impulsive processes, working memory capacity, and positive expectancies

**Figure 1:**
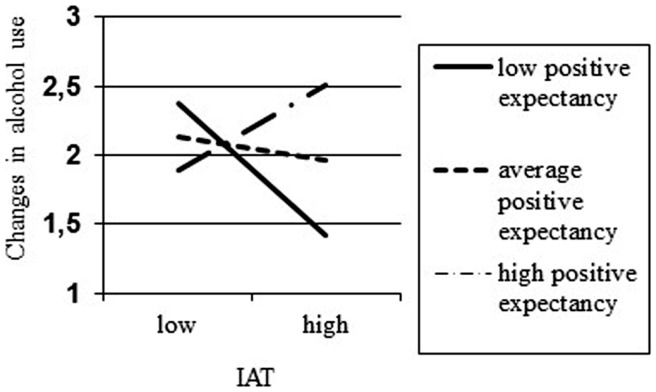
**Interaction between IAT and positive expectancies on changes in alcohol use**.

The second model included the same predictors as model 1, as well as the main effect of positive expectancies, the two-way interaction between positive expectancies and WMC, the two-way interactions between positive expectancies and the four impulsive measures, and the three-way interactions between positive expectancies, WMC, and alcohol-related impulsive processes. Changes in alcohol use were used as a dependent variable in this analysis. This model explained 26% (*p* < 0.001) of the variance of alcohol use in wave 2. Alcohol use in wave 1 and positive expectancies predicted alcohol use in wave 2. The main effect of positive expectancies was qualified by an interaction with alcohol–arousal associations. In order to interpret this interaction simple slopes were calculated for individuals with low (greater than −1 SD), medium (between −1 and 1 SD), and high ( >1 SD) values of positive expectancies [see Ref. (59)]. The results of these *post hoc* analyses are presented in Figure 1. Specifically, IAT scores were negatively associated with changes in alcohol use for adolescents with low levels of positive expectancies (*b* = −0.239, SE = 0.122, *p* = 0.050). The association between IAT scores and changes in alcohol use were not associated for adolescents with average and high levels of positive expectancies (*b* = −0.041, SE = 0.084, *p* = 0.626 and *b* = 0.157, SE = 0.137, *p* = 0.253, respectively). All other main and interaction effects were not statistically significant.

### Predicting alcohol use from alcohol-related impulsive processes, working memory capacity, and negative expectancies

**Figure 2:**
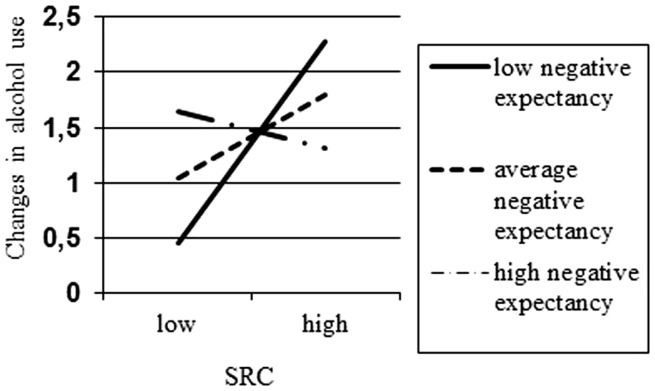
**Interaction between SRC and negative expectancies on changes in alcohol use**.

The third model also included the same predictors as model 2, but substituted the main and interaction effects involving positive expectancies with effects involving negative expectancies (*R*^2^ = 0.24, *p* < 0.001). Here too, changes in alcohol use were the dependent variable in this analysis. Aside from the main effect of alcohol use in wave 1, only one effect emerged as a statistically significant predictor: the two-way interaction between approach tendencies (SRC) and negative expectancies. Simple slopes were also calculated to interpret this interaction. Figure 2 presents the results of the association between approach tendencies and changes in alcohol use for high, medium, and low levels of negative expectancies. Specifically, SRC scores were positively associated with changes in alcohol use for adolescents with low levels of negative expectancies (*b* = 0.457, SE = 0.186, *p* = 0.014). The association between SRC scores and changes in alcohol use were not associated for adolescents with average and high levels of negative expectancies (*b* = 0.186, SE = 0.110, *p* = 0.091 and *b* = −0.085, SE = 0.121, *p* = 0.483, respectively). All other main and interaction effects were not statistically significant.

## Discussion

This study aimed to predict adolescent alcohol use by implicit cognitions (approach tendencies, attentional bias, and memory associations), explicit cognitions (expectancies), and WMC. It was hypothesized that implicit cognitions and positive expectancies would predict an increase in alcohol use, adjusting for age, gender, and previous alcohol use. Negative expectancies and WMC would predict a decrease in alcohol use.

As expected from earlier studies, positive expectancies predicted alcohol use over time [e.g., Ref. (60, 61)]. Negative expectancies did not predict changes in alcohol use. We found a similar result in a previous study of 10-year-old children, where positive expectancies predicted alcohol use, while negative expectancies and implicit associations did not (44, 50). These findings also agree with other studies showing that positive expectancies are better predictors of increases in alcohol use of adolescents [e.g., Ref. (21, 22)] and young adults (62) than implicit cognitions. This is also in line with previous research suggesting that negative expectancies start to predict alcohol use when alcohol use becomes more common practice when negative effects of alcohol have been encountered before.

Implicit cognitions, and interactions between implicit cognitions and WMC or explicit cognitions, did not consequently predict changes in alcohol use. The interaction effect between the IAT and positive expectancies showed that only in adolescents with low positive expectancies, higher IAT scores were related to less alcohol use. In addition, the interaction effect between the SRC and negative expectancies demonstrated that only in adolescents with low negative expectancies, higher SRC scores were related to more alcohol use.

It was expected that alcohol–arousal associations would be related to alcohol use (41). This study showed that male adolescents who drank alcohol associated alcohol more strongly with arousal than male adolescents who did not drink alcohol. These boys were young adolescents as well, quite similar to the age as the adolescents in the current study. Yet, the sample of adolescents from the Thush et al. (42) study reported heavier drinking patterns than our sample, which could explain the absence of an effect. Related to this issue, it was shown that heavy drinkers had an attentional bias toward alcohol use compared to adolescents who consumed less alcohol (63). In addition, a recent study reported by our lab showed that attentional bias was related to alcohol use only in carriers of the mu-opioid receptor gene (OPRM1) G-allele (51). In a study in young adult males who drank heavily, it was also shown that only OPRM1 G-allele carriers had an approach bias toward alcohol (64). From the above, we conclude that the non-significant findings from our study could be explained by the fact that the adolescents in our sample did not have enough experiences with alcohol use. Alternatively, the effects of implicit cognitions might be limited to adolescents with certain genotypes [e.g., Ref. (51)], or those with specific environmental risk factors. Based on previous studies [e.g., Ref. (42, 46)], we also expected that implicit cognitions would be more robustly related to alcohol use in adolescents with relatively poor WMC. Our findings were not in line with these studies [see also Ref. (52)] and therefore fail to support the dual process model of addiction.

Based on our findings, we argue that the dual process model of addiction may not be applicable to alcohol use in normative early adolescents. That is, the model, especially the impulsive route to alcohol use, does not seem to predict emerging alcohol use in these adolescents. There is empirical evidence for the validity of the model in predicting heavier alcohol use in adolescents, or alcohol abuse and dependence in adults. Yet, these observations do not seem to generalize to normative sample of early adolescents. Furthermore, we speculate that alcohol-induced sensitization of dopamine pathways in the brain may require more intense and frequent use. Initial and irregular alcohol use, which is so characteristic of early adolescents, may not be enough to incite sensitization.

Although this is the first longitudinal study to investigate elements of the dual process model and alcohol use in a normative sample of adolescents, it has various limitations. First, implicit cognitions, explicit cognitions, and WMC were assessed only once. As such, we were not able to examine the effect of alcohol use on subsequent implicit cognitions, explicit cognitions, and WMC. It could be that implicit cognitions do not predict increases in alcohol use yet in young adolescents, but that alcohol use does predict alterations in implicit cognitions. Nevertheless, this is beyond the scope of the current research aims. Second, our sample consisted of relatively normative adolescents, while other studies that do find associations between implicit cognitions and alcohol study less normative adolescents, e.g., from continuation high schools. At the same time, our study provides meaningful data in normative adolescents in a relatively large sample. Third, the interval between our assessments was relatively short (i.e., <1 year). Fourth, we used several indirect measures to measure implicit cognitions, but alternatives could be used in future research. In sum, future studies are warranted that employ longitudinal designs, on at-risk and normative samples to test the validity of parts of the dual process model in predicting more advanced stages of alcohol use in adolescents and young adults.

## Conflict of Interest Statement

The authors declare that the research was conducted in the absence of any commercial or financial relationships that could be construed as a potential conflict of interest.
